# Comparative transcriptomic analysis of seed coats with high and low lignin contents reveals lignin and flavonoid biosynthesis in *Brassica napus*

**DOI:** 10.1186/s12870-021-03030-5

**Published:** 2021-05-29

**Authors:** Yiran Ding, Shizhou Yu, Jia Wang, Maoteng Li, Cunmin Qu, Jiana Li, Liezhao Liu

**Affiliations:** 1 College of Agronomy and Biotechnology, Academy of Agricultural Sciences, Southwest University, Chongqing, 400715 China; 2 Guizhou Rapeseed Institute, Guizhou Academy of Agricultural Sciences, Guizhou, 550008 China; 3 Institute of Resource Biology and Biotechnology, College of Life Science and Technology, Huazhong University of Science and Technology, Wuhan 430070, Hubei, China

**Keywords:** *Brassica napus* L., Seed coat, Lignin, Flavonoid, RNA-Seq

## Abstract

**Background:**

*Brassica napus* L. (2n = 38, AACC) is one of the most important oil crops and sources of protein for animal feed worldwide. Lignin is a large molecule aromatic polymer and a major cell wall component. However, lignin in the seed coat reduces the availability and restricts the development of rapeseed cake. Therefore, it is critical to reduce the lignin content of the seed coat. Here, high-lignin (H-lignin) and low-lignin (L-lignin) content recombinant inbred lines (RILs) were selected from an RIL population for analysis.

**Results:**

The cross-section results indicated that the seed coat of the H-lignin lines was thicker than that of the L-lignin lines, especially the palisade layer. The seed coats and embryos at 35, 40 and 46 days after flowering (DAF) were subjected to RNA sequencing (RNA-Seq), and the expression of the *BnPAL* and *BnC4H* gene families in the lignin pathway was significantly higher in the H-lignin seed coat than in the L-lignin seed coat. The *Bn4CL* gene family also showed this trend. In addition, among the genes related to plant hormone synthesis, *BnaC02g01710D* was upregulated and *BnaA07g11700D* and *BnaC09g00190D* were downregulated in H-lignin lines. Some transcription factors were upregulated, such as *BnNAC080*, *BnNAC083*, *BnMYB9*, *BnMYB9-1*, *BnMYB60* and *BnMYB60-1*, while *BnMYB91* was downregulated in H-lignin lines. Moreover, most genes of the flavonoid pathway, such as *BnCHS* and *BnDFR*, were strongly expressed in H-lignin seed coat.

**Conclusions:**

In Our study, some key genes such as hormone synthesis genes, transcription factors and miRNAs related to lignin and flavonoid biosynthesis were identified. A regulatory model of *B. napus* seed coat lignin was proposed. These results provide new insight into lignin and flavonoid biosynthesis in *B. napus*.

**Supplementary Information:**

The online version contains supplementary material available at 10.1186/s12870-021-03030-5.

## Background

Rapeseed (*Brassica napus* L.) is widely used in the production of vegetable oils and biofuel, and rapeseed cake obtained after oil extraction is also used as a high-quality feed for animal breeding [1]. As the main supply of protein feed for animal husbandry, rapeseed cake is rich in nutrients and protein [2]. A moderate amount of fibre in the feed can promote digestion in animals and increase economic benefits. However, an excessive fibre content in feed could affect digestion and absorption in the animal [3] and restrict the development and availability of rapeseed cake.

Lignin is a complex biomacromolecular polymer that usually surrounds the polysaccharide components of plant cell walls, providing sufficient compressive strength to the cells to make the outer wall hydrophobic and waterproof [4–6]. Lignin appears in various tissues of plants, such as stems, roots, petioles, pods, and seed coats. There are three main types of lignin, i.e., *p*-hydroxyphenyl lignin (H-type lignin), syringyl lignin (S-type lignin) and guaiacyl lignin (G-type lignin), all of which are formed by polymerization of three monomers, *p*-coumaryl alcohol, sinapyl alcohol and coniferyl alcohol [7]. The lignin in angiosperms is mainly composed of G-type and S-type lignin, while that in gymnosperms is mainly G-type lignin [8].

In recent years, genes encoding the key enzymes in lignin synthesis have been cloned and functionally studied by a large number of researchers with the ultimate goal of altering the content or composition of lignin in plants. It was proven that a reduction in L-Phenylalanine ammonia-lyase (PAL) activity resulted in a decrease in lignin content [9]. Sewalt et al. [10] revealed that the content and composition of lignin were changed when the expression of PAL and cinnamate 4-hydroxylase (C4H) was altered in transgenic tobacco lines (*Nicotiana tabacum*). The lignin content of transgenic poplar (*Populus tremuloides* Michx.) and *Arabidopsis* lines were reduced when 4-coumarate: CoA ligase (4CL) expression was downregulated, and the G- and S-type lignin monomer ratio was also reduced [11–13]. As reported in some studies, genes encoding enzymes, including coumarate 3-hydroxylase (*C3H*) [14, 15], hydroxycinnamoyl-CoA shikimate/quinate transferase (*HCT*) [16], caffeoyl coenzyme A O-methyltransferase (*CCoA-OMT*) [17], cinnamoyl CoA reductase (*CCR*) [18, 19], ferulate 5-hydroxylase (*F5H*) [20], caffeic acid *O*-methyltransferase (*COMT*) [17] and cinnamyl alcohol dehydrogenase (*CAD*) [18, 21], have played a critical role in lignin biosynthesis. Laccase (LAC) and peroxidase (PER) have important catalytic effects in the formation of lignin polymers from lignin monomers [22–24]. In addition, NST1, NST2 and NST3 of the NAC transcription factor family regulate the formation of the *Arabidopsis* secondary wall, and as upstream regulators directly affect the downstream *AtMYB46* and *AtMYB83*, while overexpression of NSTs leads to ectopic lignification [25–27]. Except for F5H, most of the genes encoding key enzymes in the synthesis of lignin monomers are directly regulated by AtMYB58 via AC-acting elements [28]. Although many studies have been conducted on related topics, the molecular mechanisms of this phenotype remain unclear due to the genomic complexity of *B. napus* and the influence of other factors on the rapeseed seed coat [29, 30].

Reducing the lignin content in the seed coat is one of the important goals of rapeseed breeding. Previous studies have mapped candidate genes for the seed coat lignin content. Liu et al. [31] detected five and three QTLs accounting for 4.7% to 21.9% and 7.3% to 16.9% of the phenotypic variation for cellulose and hemicellulose by using a high-density SNP genetic map, and this result was supported by Wang et al. [32] observed three significant associations on A05, A09 and C05 by genome-wide association study (GWAS). To date, there is no transcriptomic information about the seed coat, especially for lines of *B. napus* with extremely different seed lignin contents. In the present study, different lines with high and low lignin contents in the seeds were screened, and high-throughput sequencing was used to identify the key genes involved in lignin synthesis in the seed coat of *B. napus*.

## Results

### Screening and quality trait analysis of H- and L-lignin lines

**Fig. 1:**
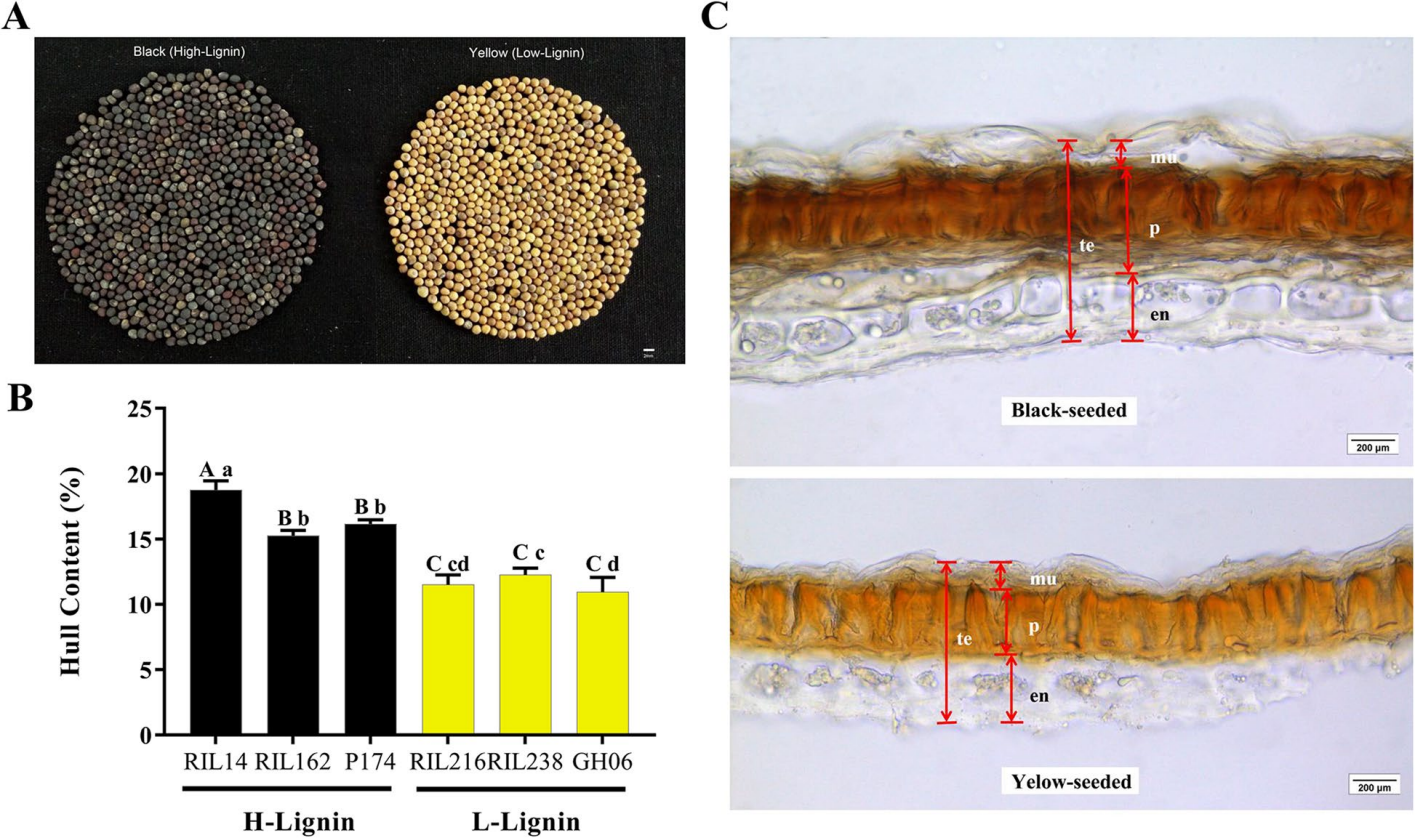
Comparison of seed colour and hull lignin content among high- (black-seeded) and low-lignin (yellow-seeded) lines. (**A**) Seed colour of H- and L-lignin lines. Bar, 2 mm. (**B**) Statistical analysis of the hull lignin contents (%) of the extreme lines. The lowercase letters and capital letters represent significance (α) at 0.05 and 0.01, respectively. (**C**) Microscopic observation of H- and L-lignin lines. Te, taste; mu, epidermal mucilage; p, palisade layer; en, endothelial layer. Bars, 200 μm

Seeds of 172 RILs of *B. napus* were harvested in May 2014 and May 2015 and scanned by a near-infrared rapid quality analyser. The ADL contents in the L-lignin lines and H-lignin lines ranged from 0.97% to 1.34% and from 2.49% to 4.70%, respectively. The L-lignin lines GH06, RIL134, RIL215, RIL216, and RIL238 and the H-lignin lines P174, RIL4, RIL14, RIL103 and RIL162 were used for subsequent comparisons (Table 1). The degree of yellowing in L-lignin lines was 116.58 to 144.71, whereas that of the H-lignin lines was 47.45 to 67.35 (Table 1). Under natural light, the coats of mature seeds in the H-lignin lines appeared greyish black, while those of the L-lignin lines appeared ginger yellow (Fig. 1A). In 2014, the average oil contents of the H- and L-lignin lines were 36.70% and 37.60%, respectively. The average oil contents of the H- and L-lignin lines were 40.99% and 42.63% in 2015, respectively, which were 4% ~ 5% higher than those in 2014. In 2014, the average protein contents of the H- and L-lignin lines were 28.25% and 30.89%, respectively. In contrast, the protein content was lower than that in 2015, and the average protein contents of the H- and L-lignin lines were 25.58% and 27.80%, respectively (Table 1). Three H-lignin (RIL14, RIL162 and P174) and L-lignin (RIL216, RIL238 and GH06) lines were chosen for comparison of hull rates. The results showed that the hull rate of H-lignin seeds was 15.26% ~ 18.76%, while that of L-lignin lines was 10.94% ~ 12.26% (Fig. 1B). The hull rate of H-lignin seeds was significantly higher than that of L-lignin lines (Table S1).

**Table 1:** Screening and quality trait analysis of H/L-lignin lines of *B. napus* L

	year 2014						year 2015					
RIL ID	ADF	ADL	NDF	Fat	Protein	Degree of yellowing	ADF	ADL	NDF	Fat	Protein	Degree of yellowing
RIL134	6.96	1.34	11.06	33.08	33.20	144.71	5.67	1.26	14.09	41.06	26.10	124.62
RIL215	5.83	1.12	9.63	38.24	30.45	118.12	5.39	1.28	8.56	38.63	30.91	126.54
RIL216	6.39	1.21	9.41	40.20	29.57	133.09	5.17	1.26	7.95	40.70	30.20	128.42
RIL238	6.19	0.96	11.48	38.70	30.04	116.58	6.20	1.19	12.04	43.28	25.86	116.56
GH06	6.25	0.97	12.05	37.77	31.18	141.84	5.69	0.94	11.54	44.46	25.94	135.70
RIL4	9.77	4.49	13.92	36.30	27.46	47.45	8.08	3.84	12.03	39.01	25.79	55.44
RIL14	9.75	4.49	14.26	35.91	27.60	59.09	8.39	3.68	14.29	38.41	27.03	78.14
RIL103	9.48	4.43	13.83	39.06	27.74	67.35	-	-	-	-	-	-
RIL162	-	-	-	-	-	-	6.86	2.49	14.26	45.23	25.82	82.06
P174	9.87	4.70	14.60	35.54	30.20	56.03	8.83	4.15	14.06	41.29	23.69	53.99

Note: "-" indicates missing data. Recombinant Inbred Line, RIL ID; Acid Detergent Lignin, ADL; Acid Detergent Fibre, ADF; Neutral Detergent Fibre, NDF

### Microscopic observation of seed coat thickness of H- and L-lignin lines

**Fig. 2:**
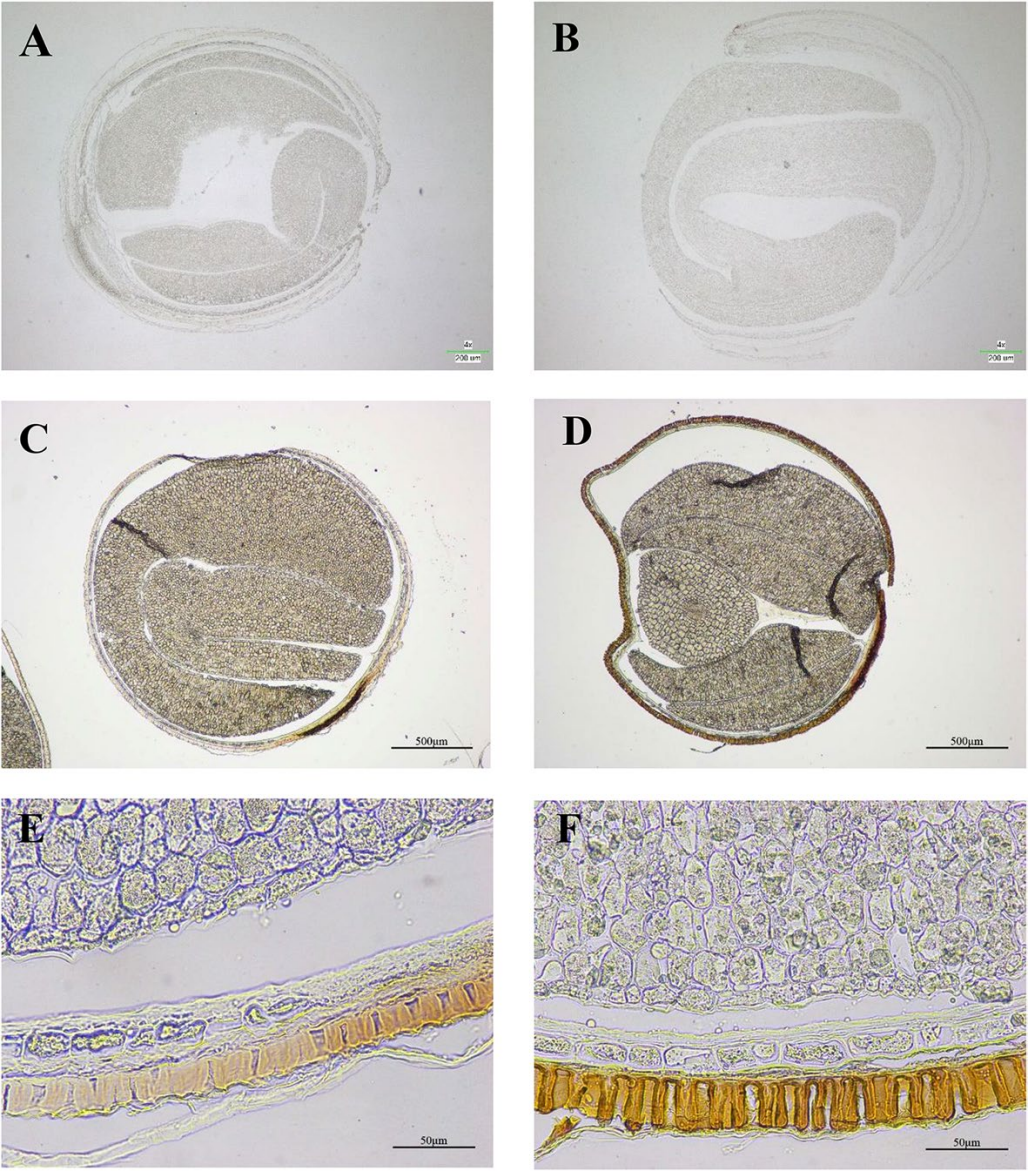
Phloroglucinol-stained sections at 40 DAF (**A**, **B**) and in mature seeds (**C**, **D**, **E**, **F**). **A**, **C**, **E**: yellow seeds, L-lignin (GH06); **B**, **D**, **F**: black seeds, H-lignin (P174). Bars, 200 μm (**A**, **B**), 500 μm (**C**, **D**, **E**, **F**)

To investigate whether the lignin content is related to the seed coat thickness, transverse sections of the seed coat of the H- and L-lignin lines (P174/GH06) were obtained for microscopic analysis. It was revealed that the palisade (p) of the H-lignin lines was thicker than that of the L-lignin lines; otherwise, the mucilaginous epidermal (mu) and endothelial layers (en) were not significantly different between the L-lignin and H-lignin lines (Fig. 1C). The results indicated that the thicker seed coat in the H-lignin line than in the L-lignin line mainly resulted from the difference in the palisade layer. Further analysis revealed that lignin was mainly distributed in the palisade of the seed coat and in small amounts in the endothelium (Fig. 2). The lignin staining in the H-lignin line seed coat was very strong (Fig. 2B, D and F), while the seed coat of the L-lignin line remained very light with staining (Fig. 2A, C and E). Because the lignin content in the seed coat is relatively low (relative to that in other tissues such as stems and roots) and the palisade of the seed coat is pigmented, it was difficult to observe dyeing.

### Transcriptomic analysis of H- and L-lignin seed coats and embryos

A total of 98.58 Gb of clean data were obtained, and the Q30 of 12 samples was ≥ 91.33% (Table S2), which verified the quality of the transcriptome sequencing data. The obtained 12 samples of clean data were compared with the *B. napus* reference genome, and the average comparison efficiency of seed coats and embryos was 71.60% and 77.52%, respectively (Table S3). A total of 15,030 DEGs were screened (the number of DEGs is the sum of each DEG set) (Table S4). With the development of the seed, the number of upregulated and downregulated genes in the seed coat increased (Fig. S1), which indicated that complex changes may occur.

### GO and TopGO enrichment analyses

The upregulated and downregulated DEGs of H- and L-lignin lines in the three seed development stages were annotated with the GO database. At 35 DAF, 1698 DEGs were annotated for "LC1 vs HC1", and more than 80% of DEGs were enriched in cell and cell parts. More than 60% of upregulated DEGs were enriched in organelles, metabolic processes and cellular processes; more than 60% of downregulated DEGs were enriched in organelles and metabolic processes (Fig. S2A). At 40 DAF, a total of 1839 DEGs were annotated for "LC2 vs HC2", and more than 80% of DEGs were enriched in cell and cell parts. More than 60% of upregulated genes were enriched in organelles, metabolic processes and cellular processes, and more than 40% of the DEGs were enriched in catalytic activity, binding and response to stimulus (Fig. S2B). At 46 DAF, 2093 DEGs were annotated for "LC3 vs HC3", and the upregulated genes were mainly enriched in cell, cell part, organelle, catalytic activity and metabolic process; the downregulated genes were mainly enriched in cell, cell part and organelle, and catalytic activity (Fig. S2C).

**Fig. 3:**
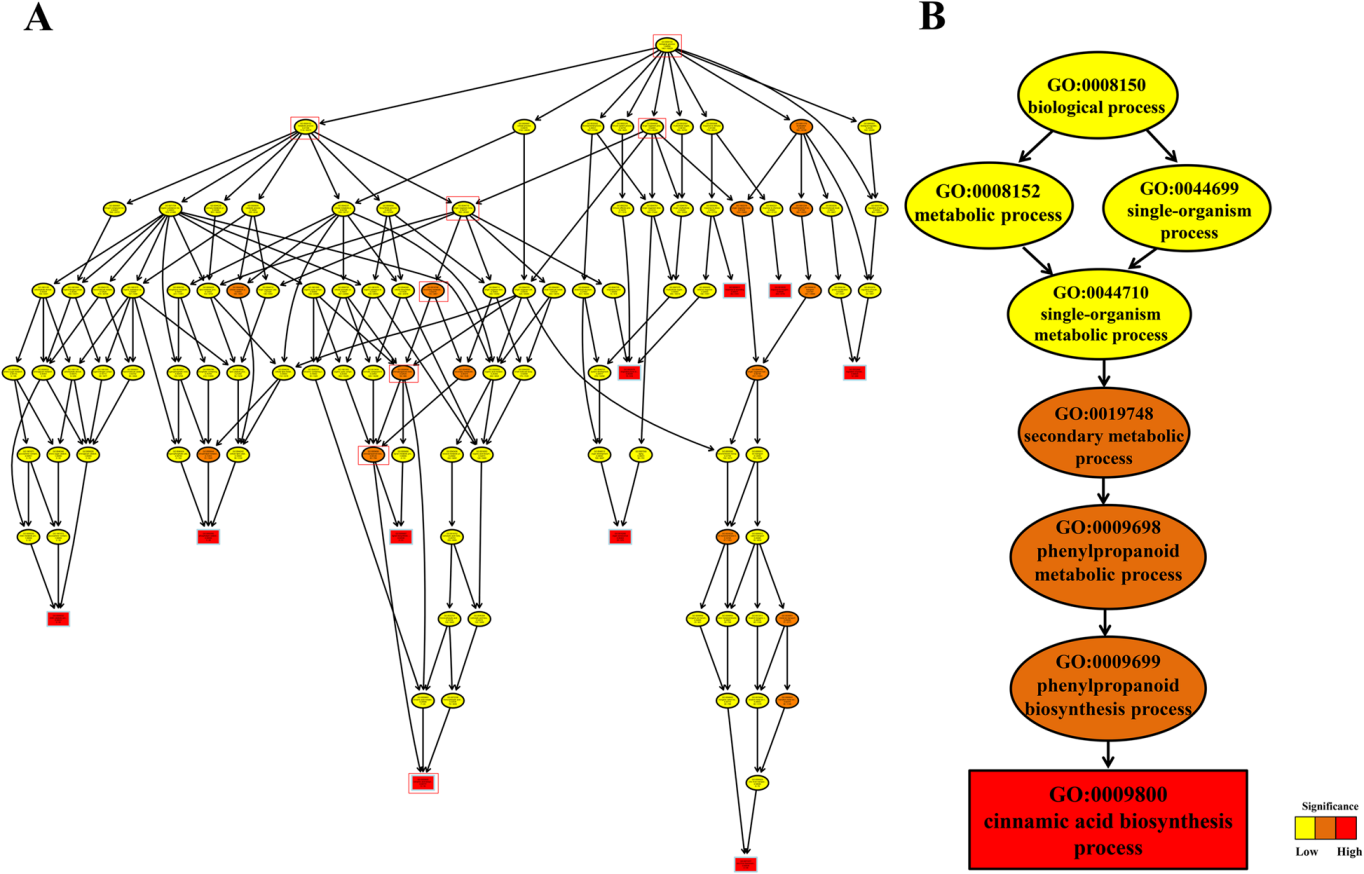
TopGO analysis of DEGs at the first stage of seed development. (**A**) All DEGs related to biological metabolic processes. (**B**) A hierarchical GO structure leading to the cinnamic acid biosynthesis process. The portion of A marked in red is detailed in **B**

TopGO analysis was performed to further explore the enrichment of DEGs in the three stages of seed development in the H- and L-lignin lines. Based on KS significance, GO terms associated with lignin biosynthesis were screened with a threshold of KS < 0.01. During seed development, DEGs associated with the "cell wall" and "secondary metabolic processes" were significantly enriched in "LC2 vs HC2" and "LC3 vs HC3" (Table 2). In addition, cinnamic acid biosynthetic process, vacuole, phenylalanine ammonia-lyase activity and trans-cinnamate 4-monooxygenase activity were significantly enriched, which indicated that these biological metabolic processes have a close relationship with lignin synthesis. The "biological process" (GO: 0008150) category of the GO primary classification contained nine biological processes that might be involved in lignin synthesis, namely, metabolic process (GO: 0008152), cellular process (GO: 0009987), developmental process (GO: 0032502), immune system process (GO: 002376), single-organism process (GO: 0044699), multicellular organismal process (GO: 0032501), response to stimulus (GO: 0050896), localization (GO: 0051179) and biological regulation (GO: 0065007) (Fig. 3A). The cinnamic acid biosynthesis process was significantly enriched, and the secondary GO terms specifically enriched were metabolic process/single-organism process, single-organism metabolic process, secondary metabolic process, phenylpropanoid metabolic process, phenylpropanoid biosynthesis process, and cinnamic acid biosynthetic process (Fig. 3B). The cinnamic acid biosynthesis process was significantly enriched, indicating that some of the genes involved in this process might promote the biosynthesis of seed coat lignin.

**Table 2:** GO terms related to lignin biosynthesis between each sample pair

GO	Term	KS	DEG set
GO:0009800	cinnamic acid biosynthetic process	0.0078	LC1 vs HC1
GO:0005618	cell wall	8.60E-05	
GO:0005773	vacuole	0.00185	
GO:0045548	phenylalanine ammonia-lyase activity	0.00013	
GO:0016710	trans-cinnamate 4-monooxygenase activity	0.00855	
GO:0019748	secondary metabolic process	0.00023	LC2 vs HC2
GO:0071554	cell wall organization or biogenesis	0.00836	
GO:0005618	cell wall	5.40E-05	
GO:0019748	secondary metabolic process	0.00066	LC3 vs HC3
GO:0005618	cell wall	0.0000056

### KEGG metabolic pathway enrichment analysis

**Fig. 4:**
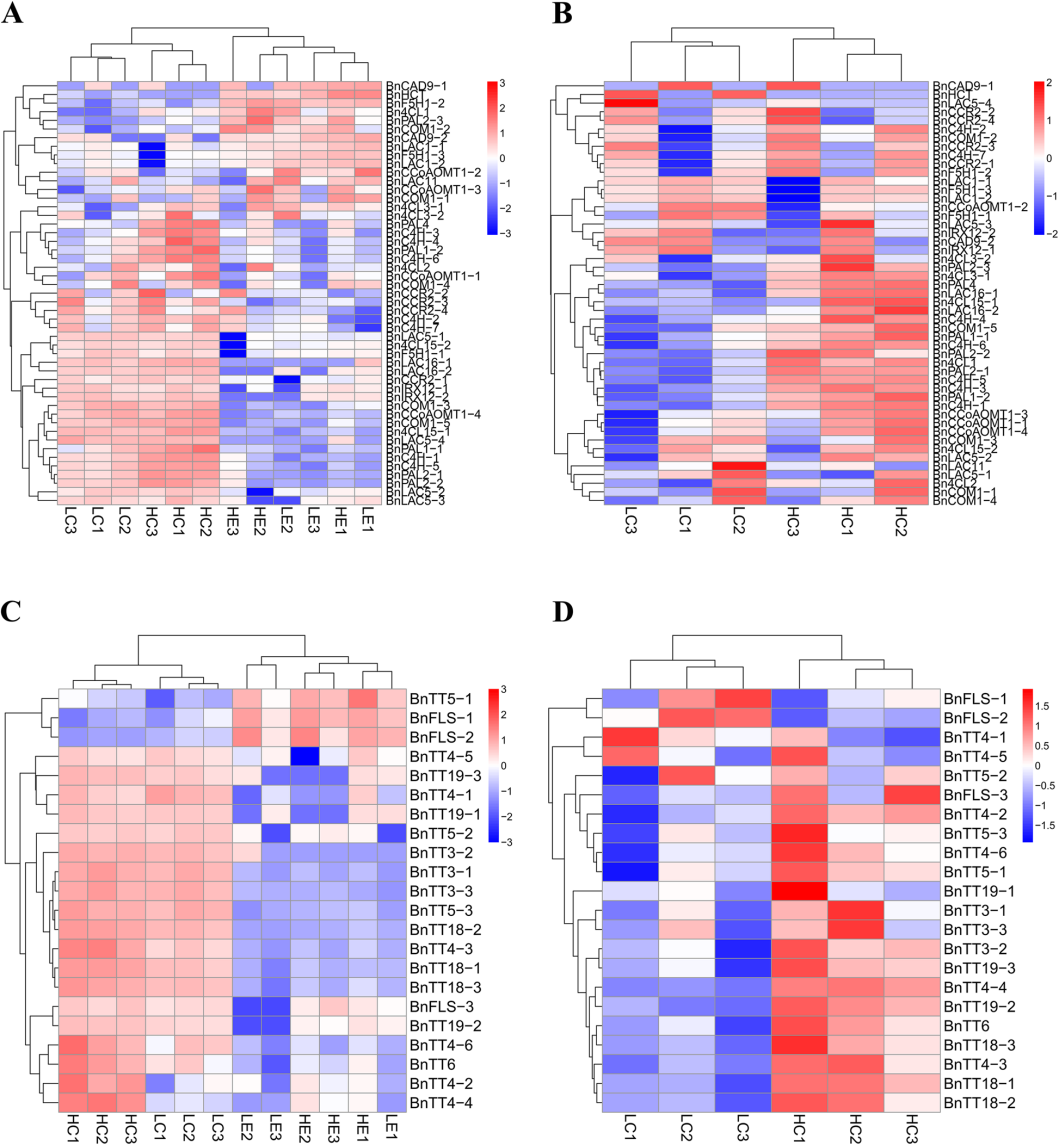
Cluster analysis of DEGs of the phenylpropanoid-lignin pathway in (**A**) 12 samples and (**B)** 6 samples from seed coats. Cluster analysis of DEGs of the flavonoid pathway in (**C**) 12 samples and (**D**) 6 samples from seed coats. Red, upregulated. Blue, downregulated

To explore the metabolic pathways enriched for the DEGs, the related DEGs in 12 samples were subjected to KEGG (Kyoto Encyclopedia of Genes and Genomes) metabolic pathway enrichment analysis. The KEGG metabolic pathways are divided into five functional regions, namely, organismal systems, metabolism, genetic information processing, environmental information processing, and cellular processes. In "LC1 vs HC1", most of the DEGs were enriched in the biosynthesis of amino acids, starch and sucrose metabolism, carbon metabolism, etc. (Fig. S3A). Most of the DEGs were enriched in ribosomes, biosynthesis of amino acids, starch and sucrose metabolism and protein processing in the endoplasmic reticulum. In "LC2 vs HC2" (Fig. S3B) and in "LC3 vs HC3", most of the DEGs were enriched in ribosome, starch and sucrose metabolism, phenylpropanoid biosynthesis and biosynthesis of amino acids. (Fig. S3C). This result indicated that the synthesis of a large number of amino acids, starch and sucrose promoted the formation of phenylpropane compounds, thereby further promoting lignin biosynthesis. Moreover, the DEGs were significantly enriched in phenylalanine metabolism, biosynthesis of amino acids and flavonoid biosynthesis during the three stages of seed development (Fig. S4). As expected, cluster analysis of the phenylpropanoid-lignin synthesis and flavonoid synthesis pathways revealed that related genes were upregulated in the seed coat and downregulated in the embryo (Fig. 4A, C), and the expression levels in the H-lignin lines (HC1, HC2 and HC3) were significantly higher than those in the L-lignin lines (LC1, LC2 and LC3) (Fig. 4B, D).

**Fig. 5:**
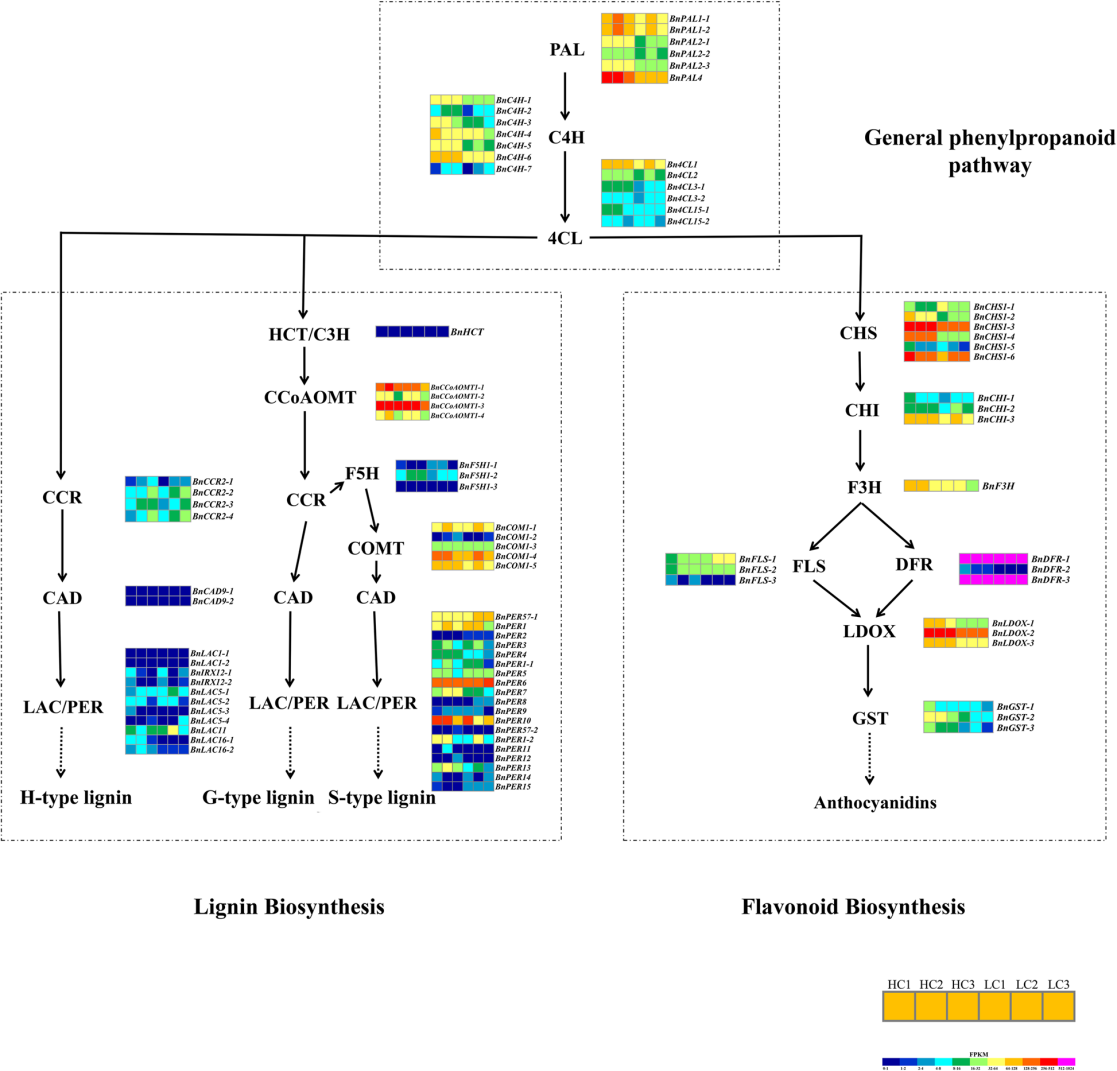
Expression analysis of key enzymes in the phenylpropane-lignin-flavonoid biosynthesis pathway. PAL, phenylalanine ammonia-lyase; C4H, cinnamate 4-hydroxylase; 4CL, 4-coumarate: CoA ligase; HCT, hydroxycinnamoyl-CoA shikimate/quinate transferase; C3H, coumarate 3-hydroxylase; CCoAOMT, caffeoyl-CoA-*O*-methyltransferase; CCR, cinnamoyl CoA reductase; F5H, ferulate 5-hydroxylase; COMT, caffeic acid *O*-methyltransferase; CAD, cinnamyl alcohol dehydrogenase; PER, peroxidase; LAC, laccase. CHS, chalcone synthase; CHI, chalcone isomerase; F3H, flavanone 3-hydroxylase; FLS, flavonol synthase; LDOX, leucoanthocyanidin dioxygenase; DFR, dihydroflavonol 4-reductase; GST, glutathione S-transferase. The colours indicate the expression levels (FPKM) of the DEGs in HCl, HC2, HC3, LC1, LC2, and LC3. The expression levels (FPKM) corresponding to the different colours are 0–16, 16–32, 32–64, 64–128, 128–256, 256–512, and 512–1024

For DEG analysis, the genes encoding PAL, C4H and 4CL in the phenylpropane pathway were highly expressed and upregulated in H-lignin lines (Fig. 5). *BnPAL4* was highly expressed in the seed coat of H-lignin lines, and the differential expression fold changes in "LC1 vs HC1" and "LC2 vs HC2" were 2.98- and 4.35-fold, respectively (Fig. S4B). Among the specific pathways for lignin synthesis, the *BnCCoAOMT* and *BnCOMT* gene families were strongly expressed in the seed coat (Fig. 5). We also observed that the genes encoding key enzymes of the flavonoid pathway were highly expressed, especially the CHS and DFR family genes. Moreover, the expression of most genes was higher in the H-lignin lines than in the L-lignin lines (Fig. 5). At 35, 40 and 46 DAF, all members of the *BnPAL* gene family and *BnC4H* gene family were significantly more highly expressed in the H-lignin seed coat than in the L-lignin seed coat (Fig. S5B, C), and most members of *Bn4CL* also exhibited the same phenomenon (Fig. S5D). *BnPAL1-1*, *BnPAL1-2*, *BnPAL4*, *BnC4H-1*, *BnC4H-2* and *BnC4H-5* all showed the highest expression at 40 DAF and a decrease in the expression at 46 DAF in the seed coat of H-lignin lines; in the L-lignin lines, *BnPAL1-1*, *BnPAL1-2*, *BnPAL2-1*, *BnPAL2-2*, *BnC4H-1*, *BnC4H-3*, *BnC4H-4*, *BnC4H-5*, *BnC4H-6* and *Bn4CL1* exhibited the highest expression at 40 DAF and decreased expression at 46 DAF (Fig. S5B, C and D).

**Fig. 6:**
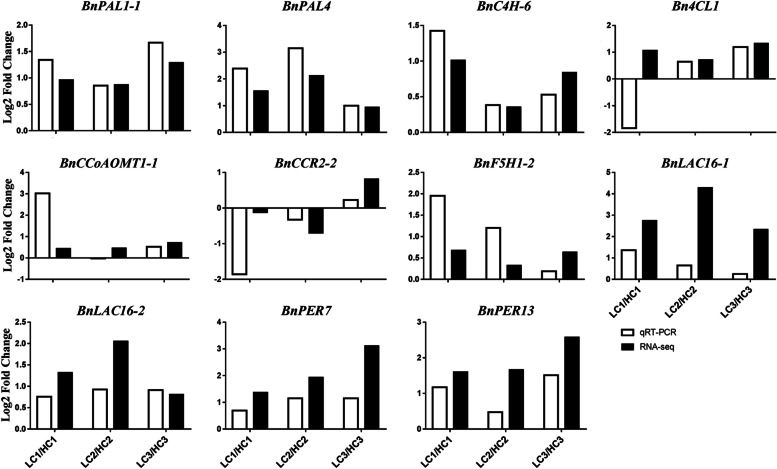
Validation of lignin pathway-related DEGs by qRT-PCR

To further verify the expression of these key genes related to lignin biosynthesis in the seed coat of *B. napus*, 22 genes, including 20 upregulated and two downregulated genes, were selected for qRT-PCR analysis (Table S5). The gene expression levels (log_2_FC) by qRT-PCR were consistent with those by RNA-Seq (Figs. 6, S6), and the reliability of the data provided by RNA-Seq was confirmed.

### Transcription factor and plant hormone analyses

**Fig. 7:**
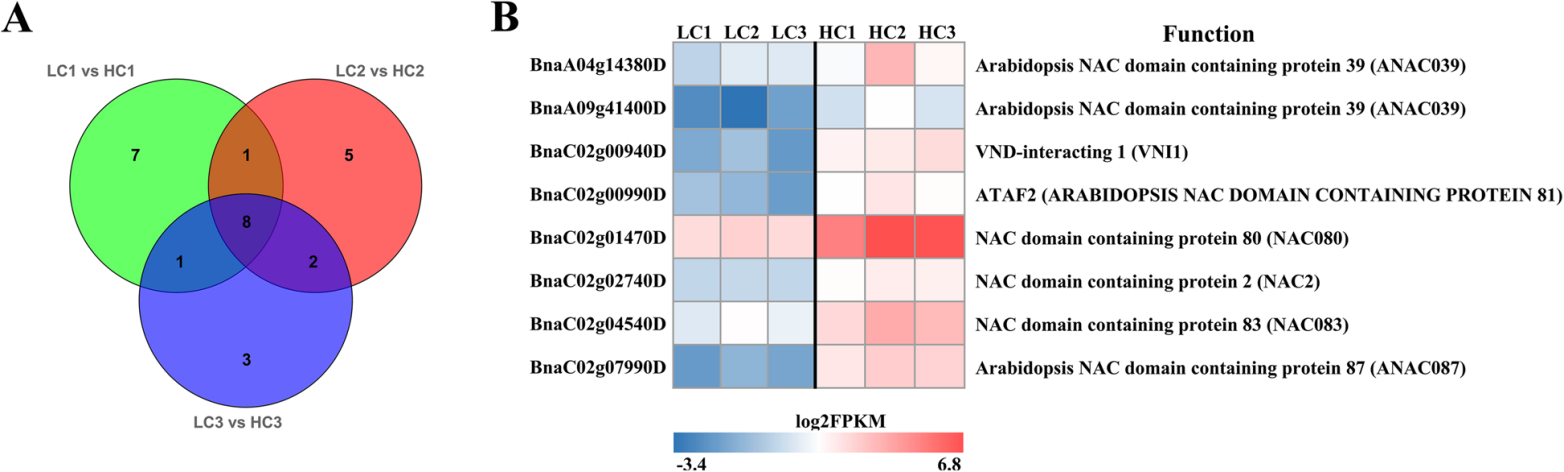
Analysis of NAC genes in three DEG sets. (**A**) Venn diagram of DEGs encoding NACs in three DEG sets. (**B**) Analysis of coexpressed genes in three DEG sets

The NAC, bZIP and ERF families were the top three differentially expressed transcription factor families in "LC1 vs HC1", with 17 (12 up- and 5 downregulated), 11 (2 up- and 9 downregulated) and 10 (4 up- and 6 downregulated) DEGs, respectively (Fig. S7A). In "LC2 vs HC2", the top three differentially expressed transcription factor families were 16 NACs (12 up- and 4 downregulated), 13 bZIPs (3 up- and 10 downregulated) and 10 MYBs (6 up- and 4 downregulated) (Fig. S7B). The top 4 differentially expressed transcription factors in "LC3 vs HC3" included 13 bZIPs (4 up- and 9 downregulated), 11 bHLHs (4 up- and 7 downregulated), 11 MYBs (9 up- and 2 downregulated) and 11 upregulated NACs (Fig. S7C). It is interesting that the number of upregulated NAC transcription factors was greater than the number of downregulated transcription factors in the three stages of seed development. Eight members of the NAC transcription factor family showed different expression in all three stages of seed development, and all of them were upregulated (Fig. 7A). Specifically, *BnNAC080* (NAC domain-containing protein 80) and *BnNAC083* (NAC domain-containing protein 83) showed the highest differential expression fold changes (Fig. 7B). Their upregulated expression might regulate downstream structural genes of lignin synthesis. According to the differential expression fold changes (Log_2_FC) and expression levels (FPKM), five MYB transcription factors were screened, including *BnMYB9* (*BnaA03g06190D*), *BnMYB60* (*BnaA08g26750D*), *BnMYB60-1* (*BnaCnng06440D*) and *BnMYB9-1* (*BnaC09g41070D*), which were upregulated, and *BnMYB91* (*BnaC03g20830D*) which was downregulated (Table S7). *BnMYB9*, *BnMYB60*, *BnMYB60-1* and *BnMYB91* were differentially expressed in the H- and L-lignin lines at 40 and 46 DAF, whereas *BnMYB9-1* was differentially expressed in the H- and L-lignin lines at 35 and 46 DAF. Based on the analysis of differential expression fold changes and expression patterns, this study screened for key genes encoding phytohormone synthesis. *BnaC02g01710D* and *BnaC09g00190D* were up- and downregulated, respectively, during the three stages of seed development in the H-lignin lines, whereas *BnaA07g11700D* was upregulated only at 35 and 46 DAF in the H-lignin lines (Table S1).

According to the prediction of miRNAs, the *BnPAL*, *BnC4H*, and *Bn4CL* genes were regulated by 25, 12, and 24 miRNAs, respectively (Fig. S8A). bna-miR172c, bna-miR396a and bna-miR172b regulated the most genes; bna-miR172c negatively regulated *BnC4H-3*, *BnC4H-5*, *Bn4CL1* and *Bn4CL5*, bna-miR396a negatively regulated *BnPAL1-1*, *BnPAL1-2* and *Bn4CL5*, bna-miR172b negatively regulated *BnC4H-3, BnC4H-5*, *Bn4CL1*, and *Bn4CL5*, and *BnPAL4* and *BnC4H-2* were only regulated by bna-miR6034 and bna-miR6033, respectively (Fig. S8B).

### Identification of candidate genes by combining GWAS and RNA sequencing

**Fig. 8:**
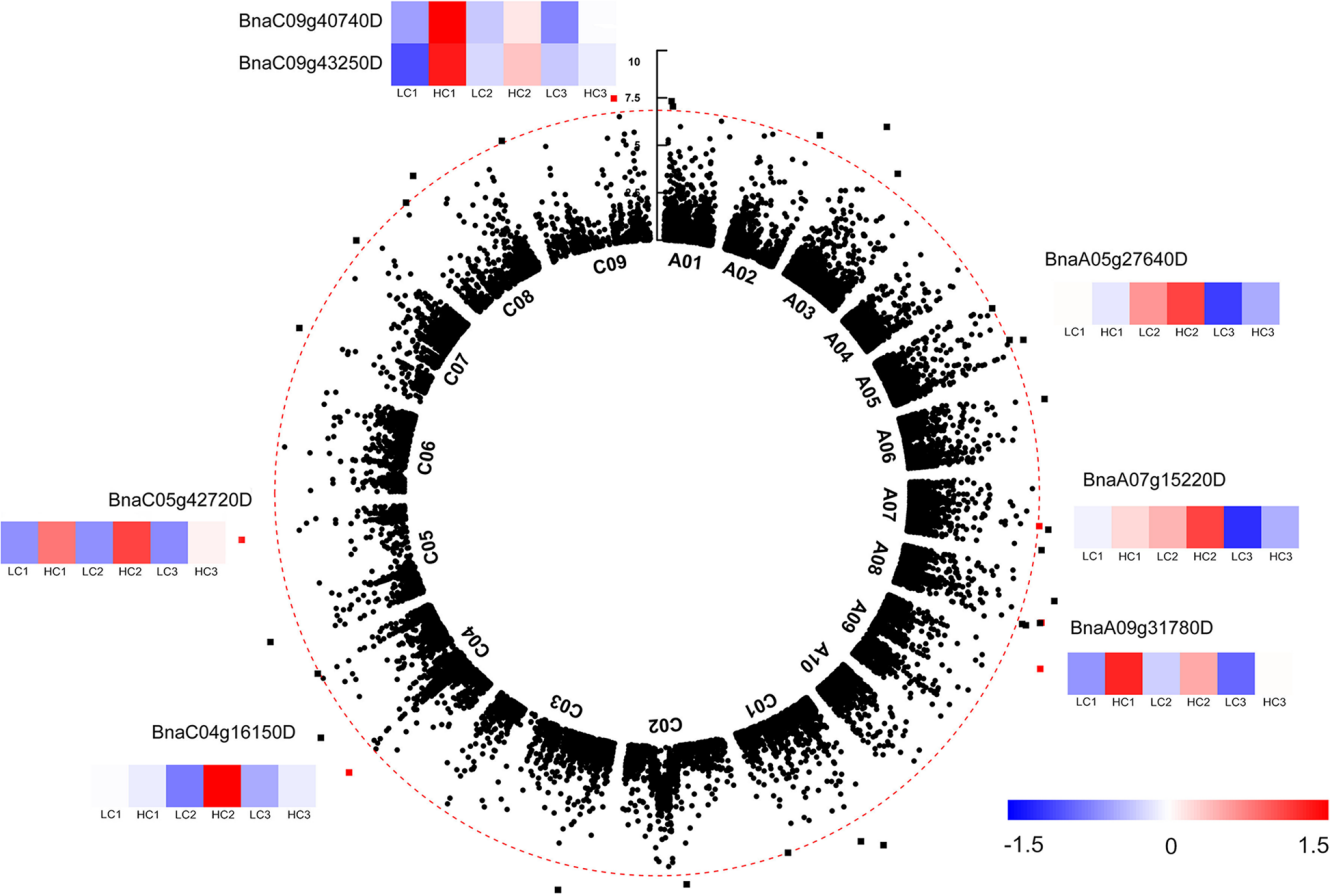
Identification of candidate seed coat lignin content genes by combined RNA sequencing with GWAS

To identify potential candidate genes related to seed coat lignin content, we systematically explored the DEGs between H- and L-lignin lines. In total, 4270 significantly DEGs were identified (Fig. S9). For the seed coat hull lignin content, 123 candidate genes were detected by GWAS in a previous report [32]. Among the candidate genes, seven genes, *BnaA05g27640D*, *BnaA07g15220D*, *BnaA09g31780D*, *BnaC04g16150D*, *BnaC05g42720D*, *BnaC09g40740D*, and *BnaC09g43250D*, were confirmed by RNA sequencing analyses (Fig. 8).

### Possible network of seed coat lignin in *B. napus*

**Fig. 9:**
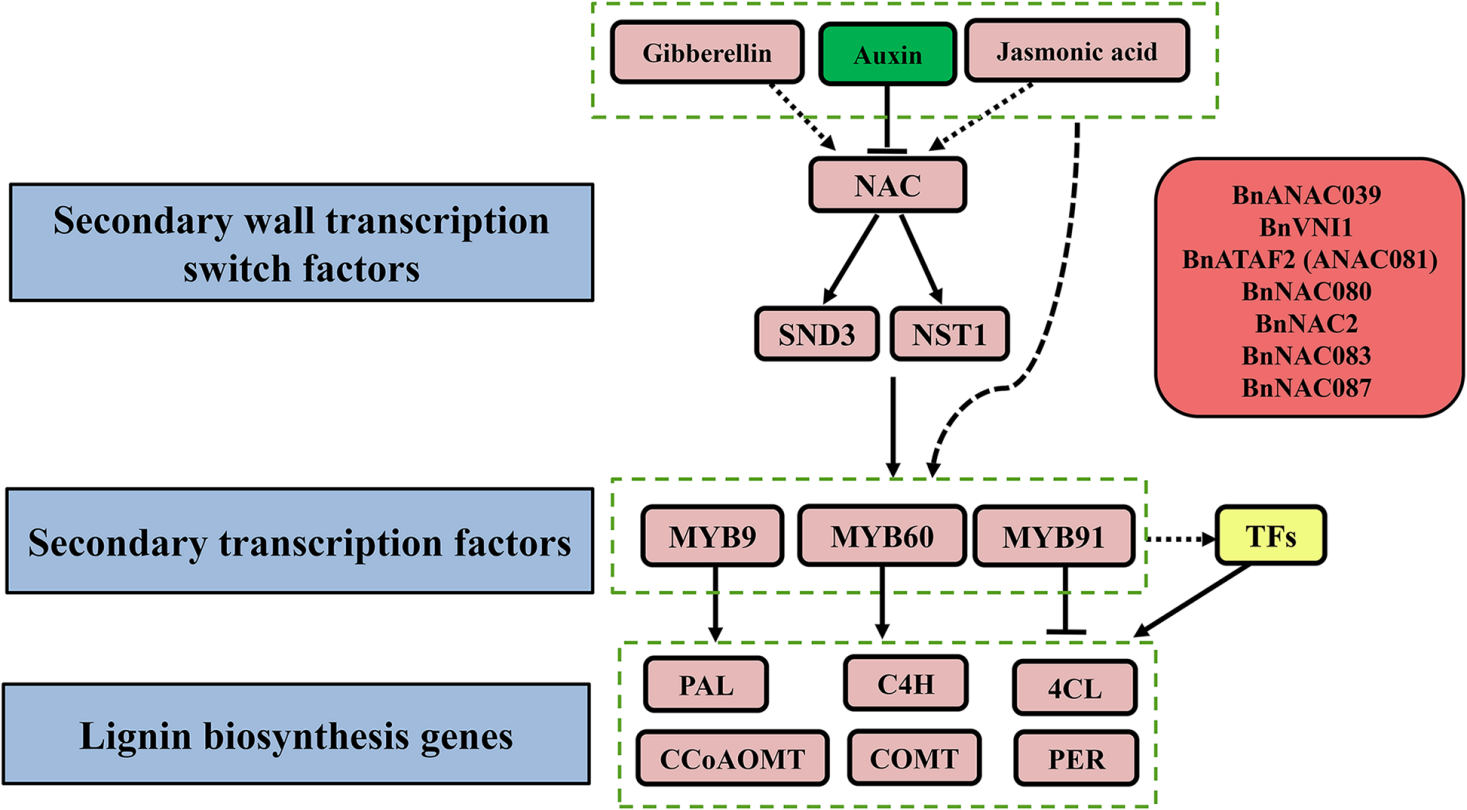
Possible model of lignin regulation in the seed coat of *B. napus* L

The regulatory network of *B. napus* seed coat lignin was drawn with the combination of the present transcriptome data and that from previous studies [33, 34]. The results are shown in Fig. 9. The NAC domain transcription factors play a "master switch" role in the regulation of the secondary wall and further regulate downstream MYB9, MYB60 and MYB91 through SND3 and NST1, while the MYB domain transcription factors are a "secondary switch". MYB9 and MYB60 positively regulate the structural genes of downstream lignin-specific synthesis, whereas MYB91 negatively regulates the structural genes. Moreover, MYB91 may also regulate other transcription factors. NAC domain transcription factors may also be actively affected by gibberellins and jasmonic acid, and are negatively affected by auxin. In addition, gibberellins, jasmonic acid and auxin may also act directly on MYB domain transcription factors.

## Discussion

### Lignin content and seed coat thickness

The seed coats of yellow-seeded *B. napus* lines are thin, and the proportion occupied by the embryo is large, thereby increasing the contents of oil and protein in the same genetic background [35]. The seed lignin content is significantly negatively correlated with the seed oil content [36]. The hull rate of yellow- and black-seeded *B. napus* is different at different developmental stages. In this study, the hull rate of the H-lignin lines was significantly higher than that of the L-lignin lines, which was consistent with the results of Wang et al. [32]. To observe the thickness of the seed coat, seed coat cross sections were observed microscopically. The results suggested that the mature rapeseed seed coat was roughly divided into three layers, the mucous epidermal cells and the palisade and endothelial layers, from outside to inside. The seed coat of the H-lignin lines was obviously thicker than the seed coat of L-lignin lines, and this was mainly dependent on the difference in the thickness of the palisade layer. Previous reports have shown that the palisade layer of the seed coat is developed from the endothelial layer of the seed [37], pigment accumulates in the palisade layer, and the black seed pigment is significantly more abundant than the yellow seed pigment. We proposed that lignin was deposited in the palisade cells, which increased the thickness of the cell wall, thereby increasing the overall thickness of the palisade layer.

### Effect of transcription factors on seed coat lignin

Related reports indicate that transcription factors play an important role in plant secondary walls [33, 34]. In this study, the DEGs at the three stages of seed development were annotated into the plant transcription factor database, and 120, 135 and 136 DEGs were screened and matched to 28, 34 and 34 families of transcription factors in the "LC1 vs HC1", "LC2 vs HC2" and "LC3 vs HC3" comparisons, respectively. The number of MYB and NAC transcription factors associated with lignin biosynthesis was higher, and the number of upregulated NAC transcription factors was greater than that of downregulated transcription factors. NST1, NST2 and NST3 of the NAC transcription factor family regulate the formation of the *Arabidopsis* secondary wall, and as upstream regulators directly affect downstream *AtMYB46* and *AtMYB83*, overexpression of NSTs leads to ectopic lignification [25]. Miao et al. [38] showed that MYB46 interacted with four target genes, MYB83, CCR1, 4CL1, and PAL1. In this study, eight NAC transcription factors showed upregulated expression. In particular, *BnNAC080* and *BnNAC083* showed high differential expression fold changes, and their upregulated expression may regulate structural genes of lignin biosynthesis. Zhou et al. (2009) observed that AtMYB58 regulates most of the genes encoding key enzymes in the synthesis of lignin monomers through AC-acting elements [28]. In this study, five MYB transcription factors related to lignin synthesis were screened. In the H-lignin lines, *BnMYB9, BnMYB60, BnMYB60-1* and *BnMYB9-1* were upregulated, while *BnMYB91* was downregulated.

### Effect of hormone regulation on seed coat lignin

In this study, DEGs encoding plant hormones were annotated with the plant hormone database and were associated with auxin, brassinolide, cytokinin, ethylene, gibberellin and jasmonic acid, which indicated that these hormones play a role in the regulation of seed coat lignin. Analysis of differential expression fold changes and expression patterns suggested that upregulation of *BnaC02g01710D* and *BnaA07g11700D* may promote the synthesis of lignin in the seed coat, whereas downregulation of the *B. napus* auxin pathway may have an inhibitory effect on the regulation of lignin. Cecchetti et al. (2013) showed that exogenous application of auxin can decrease the expression level of *AtMYB26* and inhibit secondary wall deposition, indicating that auxin is a negative regulator of lignin biological regulation [39], which is consistent with the results of our study.

Gibberellin 20-oxidase (GA20ox) is a key enzyme in the anabolism of gibberellin in plants [40]. The upregulated expression of *BnaC02g01710D* may increase the content of gibberellin and promote the formation of lignin in the seed coat. In the xylem of *Coleus blumei*, GA3 and low concentrations of IAA promote an increase in S-type lignin content [41]. Jasmonic acid affects lignin biosynthesis at the transcriptional level by *AtMYB61* [42–44]. In this study, the upregulated expression of *BnaA07g11700D* might promote the biosynthesis of lignin.

### Association between seed lignin and flavonoid biosynthesis

In plants, since lignin monomers are derived from the same sources of flavonoid biosynthesis, the biosynthesis of lignin and flavonoids is jointly affected by upstream phenylalanine anabolism [9]. In *Arabidopsis*, silencing *AtHCT* leads to a decrease in biomass, which has a major impact on the growth of the plant. In HCT-silenced plants, inhibition of lignin synthesis leads to conversion to flavonoids by metabolic flux reversal of chalcone synthase activity [45]. In this study, in the common phenylpropanoid pathway, the expression levels of DEGs encoding the enzymes PAL, C4H and 4CL were higher in the seed coat of H-lignin lines than in that of L-lignin lines. Similar results have been found by Jiang et al. (2019), which suggests that most DEGs are downregulated in yellow-seeded *B. napus* [46], and these results are consistent with the decreased flavonoid and lignin contents. 4CL catalyses the formation of *p-*coumaroyl-CoA by *p*-coumaric acid, which is the precursor of lignin and flavonoid biosynthesis. In the H-lignin lines, the expression of genes encoding PAL, C4H, and 4CL was significantly higher than that in the L-lignin lines. Among the specific pathways for lignin synthesis, CCoA-OMT catalyses the conversion of caffeoyl-CoA to feruloyl-CoA, which is the first methyltransfer reaction and is mainly involved in the synthesis of G-type lignin monomers. In addition, the expression of *COMT* was also high, and we speculated that lignin in the seed coat was mainly composed of G-type and S-type lignin monomers. Qu et al. (2013) found that the flavonoid content in the seed coat of *B. napus* was the highest at 42 d after pollination, and black-seeded *B. napus* had a significantly higher lignin content than yellow-seeded *B. napus* [47], which is consistent with the transcriptome data of this study. In the flavonoid synthesis pathway, the upregulated expression of DEGs encoding most of the enzymes in the H-lignin lines indicated that these enzymes have higher activity and therefore contribute to high flavonoid content in these lines. Liu et al. [31] constructed a high-density genetic linkage map for a *B. napus* recombinant inbred line population using a genome-wide single nucleotide polymorphism marker determined by a 60 K chip for four different environmental lines of *B. napus*. QTL analysis of the cell wall fibre components ADL, cellulose and hemicellulose showed that the seed colour and fibre traits of 11 QTLs were detected. The SNP sites of ADL traits are mainly located on chromosomes A09 and C05. Similar results have been found, for example, *Bna.CCR1* is localized in silico near the peak of a corresponding seed fibre QTL [48]. In addition, Wang et al. [32] performed a GWAS analysis on the seed ADL content of *B. napus*, detected significant SNPs on A05, A09 and C05 and proposed the key genes *BnaA.PAL4*, *BnaA.CAD2*/*BnaA.CAD3* and *BnaC.CCR1* for lignin biosynthesis [32]. The comparison of the GWAS and our transcriptome results revealed that *BnPAL4*, which was verified by qRT-PCR, is the key gene for the difference in the lignin content among the lines.

## Conclusions

Conclusively, our study revealed the key genes related to lignin biosynthesis in the *Brassica napus* seed coat. First, the seed coat of the H-lignin lines was thicker than that of the L-lignin lines, especially with regard to the palisade layer. Then, KEGG and GO analyses suggested that phenylpropanoid and flavonoid biosynthesis were significantly enriched in the H-lignin lines. The genes *BnPAL*, *BnC4H* and *Bn4CL* of the lignin biosynthesis pathway, *BnCHS* and *BnDFR* of the flavonoid biosynthesis pathway, *BnaC02g01710D*, *BnaA07g11700D* and *BnaC09g00190D* of plant hormone synthesis pathways, and some transcription factors *BnNAC080*, *BnNAC083*, *BnMYB9*, *BnMYB9-1*, *BnMYB60*, *BnMYB60-1* and *BnMYB91* were screened in the current study. Finally, by combined analysis of RNA-Seq and reported data, a model of *B. napus* seed coat lignin was proposed.

## Methods

### Plant materials

The seeds of two lines GH06 and P174 were observed by our lab. The population of 172 RILs was derived by single seed descent from F_2_ offspring of a cross between the Chinese semi-winter rapeseed parental lines GH06 (yellow seeds, low lignin content) and P174 (black seeds, high lignin content) [31]. A total of 172 *B. napus* recombinant inbred lines (RILs) were grown in 3 replications under natural growth conditions for two years (September 2013 to May 2014 and September 2014 to May 2015) at the experimental farm of Southwest University, Chongqing, China.

The selected five H-lignin lines and five L-lignin lines were sown in September 2016 at the experimental farm of Southwest University (Chongqing, China) and planted under natural growth conditions. A randomized complete block design was used, and the experiment was repeated 3 times. Each line was planted in two rows of 10 plants each with a line spacing of 30 cm and a distance of 20 cm between each plant. Seeds of five lines with H- and L-lignin were collected 35, 40 and 46 days after flowering (DAF) with three replications, and the seed coat and embryo were separated. The samples were immediately frozen in liquid nitrogen and stored at -80 °C for RNA-Seq. LC1, LC2 and LC3 represent low-lignin seed coats at 35, 40 and 46 DAF, respectively; HC1, HC2 and HC3 represent high-lignin seed coats at 35, 40 and 46 DAF, respectively; LE1, LE2 and LE3 indicate low-lignin embryos at 35, 40 and 46 DAF; HE1, HE2 and HE3 represent high-lignin embryos at 35, 40 and 46 DAF, respectively.

### Data analysis

Analyses of the means from three individual experiments and determination of significant differences by Duncan's multiple range test were performed with SPSS 17.0. The level of significance (α) was set at 0.01 and 0.05.

### Determination of lignin, fatty acid and protein contents

Seed traits were determined using a near-infrared rapid quality analyser (NIR System 6500, FOSS, Sweden) according to the method of Dimov et al. [49]. The instrument was warmed up for at least 50 min before scanning. A seed quality near-infrared model was used to determine seed quality traits, including the contents of acid detergent lignin (ADL), acid detergent fibre (ADF), neutral detergent fibre (NDF), fat, and protein, and the degree of yellowing in seeds.

### Determination of the hull rate

Seeds of the H- and L-lignin lines were dried in an electric thermostat-controlled blast drying oven at 80 ± 2 °C for 72 h and weighed, and the total dry weight (W) (g) of the seeds was recorded. Each seed sample was approximately 3.00 g, and 3 replicates were used. The weighed seeds were immersed in sterile water for 24 h until fully imbibed, and the seed coat and embryos were separated by using anatomic needles. The seed coat was dried in an electric thermostat blast oven at 80 ± 2 °C for 48 h, and the dry weight of the seed coat (W1) (g) was recorded. The formula was as follows:

$$Hull rate \left(\%\right)=\frac{W1}{W}\times 100\%$$

### Microscopic observation of seed coat thickness and staining of rapeseed

At 40 DAF, mature seeds were used for the observation of seed coat thickness according to the method of Zhou et al. [28]. A cryostat (CM1850UV Leica) was used for UV disinfection, and the chassis temperature was decreased to -20 °C before use. Embedding and sectioning: The embedding agent was added dropwise until the rapeseed seeds were completely covered and the colour of the embedding agent changed from transparent to white. The temperature of the cryostat cabinet was adjusted from -20 °C to -10 °C for more than 30 min. The sample holder was attached to the sample head on the microtome, and the section thickness was adjusted to 1 μm. The clean slides were placed flat on top of the cut tissue sheets to attach the tissue to the slides, and the prepared sections were placed under a microscope (Nikon SMZ1500) for observation. Sections from seeds at 40 DAF and mature seeds were stained with phloroglucinol-HCl as described by Zhou et al. [28]. The sections were transferred to glass slides, 1 mL of 1% phloroglucinol solution was added for 5 min, and then 1 mL concentrated hydrochloric acid solution was added. The sections were observed under a microscope (Nikon ECLIPSE E600) with 4 × , 10 × , 20 × , 40 × and 100 × objective lenses, and high-resolution photographs were taken.

### RNA sequencing and analysis

Total RNA was extracted from rapeseed coats and embryos of five lines at 35, 40 and 46 DAF using EZ-10 RNA Miniprep Kits (Sangon Biotech Co., Ltd., Shanghai, China). The Agilent Bioanalyzer 2100 system (Agilent Technologies, CA, USA) was used to assess RNA integrity. High-quality RNA from all three samples was obtained by Biomarker Technologies Co., Ltd. (Beijing, China) for RNA sequencing and analysis. TopHat2 software was used for reference genome (*Brassica napus* v4.1) mapping.

Fragments per kilobase of exon per million fragments mapped (FPKM) values were used to represent the levels of gene expression. The EBSeq R package was used to analyse the differentially expressed genes (DEGs) with an false discovery rate (FDR) < 0.01 and |log_2_(fold change)|≥ 1. Two samples were compared using the "A vs B" method, wherein the "A" sample was used as a control. If the expression level of a DEG in sample "B" was higher than that in "A", the DEG was upregulated; otherwise, it was downregulated. We used the GOseq R package [50] to perform GO enrichment analysis of the DEGs, and DEG enrichment in KEGG pathways was assessed using KOBAS software [51]. In this study, DEGs of the three sets of comparisons ("LC1 vs HC1", "LC2 vs HC2" and "LC3 vs HC3") were annotated in Plant Transcription Factor Database v4.0 [52] and *Arabidopsis* Hormone Database 2.0 [53]. The raw RNA-Seq data have been uploaded to NCBI under the accession number PRJNA597958.

### Prediction of miRNAs targeting the *BnPAL*, *BnC4H* and *Bn4CL* genes

The genome sequences of *BnPAL*, *BnC4H* and *Bn4CL* family genes were submitted as candidate genes for predicting potential microRNAs (miRNAs) by searching against the available *B. napus* reference miRNA sequences by using a plant small RNA target analysis server (psRNATarget) [54]. Cytoscape software was used to analyse the interactions between miRNAs and corresponding target genes.

### Validation of RNA-Seq data

A quantitative reverse-transcription PCR (qRT-PCR) experiment was performed to verify the differential expression levels of DEGs measured by RNA-Seq according to Zhao et al. [55] and Zhang et al. [56]. Primers were designed using Primer Premier 5 and synthesized by Sangon Biotech (Shanghai) Co., Ltd. (Shanghai, China). Primer Premier 5.0 software was used to design specific primers for quantitative RT-PCR (Table S5), and qPCR was performed using a Bio-Rad CFX96 Real-time System with SYBR® Green PCR Supermix (California, USA). Each reaction contained 10 μL of SYBR Supermix, 0.4 μL of each primer (10 μM), 7.2 μL of H_2_O, and 2 μL of cDNA in a final volume of 20 µL. Three technical replicates were used for each reaction. The following procedure was used for qRT-PCR: 98 °C for 30 s, followed by 40 cycles of 98 °C for 10 s and 60 °C for 30 s. The *BnActin7* gene was used as a control. To verify the expression levels detected by RNA-Seq, the RNA-Seq data were compared to the data obtained by qRT-PCR.

## Supplementary Information

**Additional file 1:** **Table S1.** Variance analysis of the hull lignin content in *B. napus* L."*" significant at *P*  <  0.05. **Table S2.** Quality of the sequencing data. **Table S3.** Alignment efficiency statistics of the sequencing data. **Table S4.** Numbers of DEGs. **Table S5.** Primers used for real-time quantitative PCR. **Table S6.** The key DEGs related to plant hormone synthesis between each sample pair. **Table S7.** The key DEGs encoding MYB TFs between each sample pair.

**Additional file 2: Fig. S1.** Numbers of DEGs at three stages of seed coat development in H- and L-lignin lines. Red, upregulated; black, downregulated.

**Additional file 3: Fig. S2.** GO enrichment analysis of DEGs in three DEG sets: (A) LC1 vs HC1, (B) LC2 vs HC2 and (C) LC3 vs HC3.

**Additional file 4: Fig. S3.** KEGG analysis of DEG sets: (A) LC1 vs HC1, (B) LC2 vs HC2, and (C) LC3 vs HC3.

**Additional file 5: Fig. S4.** Top 20 significantly enriched KEGG terms of DEGs in three sets: (A) LC1 vs HC1, (B) LC2 vs HC2 and (C) LC3 vs HC3.

**Additional file 6: Fig. S5.** Expression of DEGs encoding PAL, C4H, and 4CL in the phenylpropanoid pathway during three stages of seed coat development. (A) Number of DEGs in the *BnPAL*, *BnC4H*, and *Bn4CL* gene families. The expression (FPKM) of (B) *BnPAL*, (C) *BnC4H* and (D) *Bn4CL* gene families in three developmental stages of H- and L-lignin seed coats.

**Additional file 7: Fig. S6.** Validation TFs and hormones related to lignin biosynthesis by qRT-PCR.

**Additional file 8: Fig. S7.** Numbers of DEGs encoding TFs at three stages of seed coat development in H- and L-lignin lines. (A) LC1 vs HC1 (B) LC2 vs HC2 (C) LC3 vs HC3.

**Additional file 9: Fig. S8.** Related miRNAs of BnPAL, BnC4H and Bn4CL gene families. (A) Whole miRNAs of *BnPAL*, *BnC4H* and *Bn4CL* gene families. (B) MiRNAs with the largest number of regulatory genes.

**Additional file 10: Fig. S9.** Overlapping DEGs in the seed coat transcriptome and previous GWAS studies.

## Data Availability

The datasets generated and analyzed during the present study are available from the corresponding author on reasonable request. All sequencing data have been deposited in SRA (www.ncbi.nlm.nih.gov/sra). The accession number is PRJNA597958 available at https://www.ncbi.nlm.nih.gov/sra/.

## Abbreviations

H-lignin: High-lignin
L-lignin: Low-lignin
DAF: Days after flowering
RNA-Seq: RNA sequencing
H-type lignin: *p*-Hydroxyphenyl lignin
S-type lignin: Syringyl lignin
G-type lignin: Guaiacyl lignin
PAL: L-Phenylalanine ammonia-lyase
C4H: Cinnamate 4-hydroxylase
4CL: 4-Coumarate: CoA ligase
C3H: Coumarate 3-hydroxylase
HCT: Hydroxycinnamoyl-CoA shikimate/quinate transferase
CCoA-OMT: Caffeoyl coenzyme A O-methyltransferase
CCR: Cinnamoyl CoA reductase
F5H: Ferulate 5-hydroxylase
COMT: Caffeic acid O-methyltransferase
CAD: Cinnamyl alcohol dehydrogenase
LAC: Laccase
PER: Peroxidase
GWAS: Genome-wide association study
KEGG: Kyoto Encyclopedia of Genes and Genomes
GA20ox: Gibberellin (GA) 20-oxidase
RILs: Recombinant inbred lines
ADL: Acid detergent lignin
ADF: Acid detergent fibre
NDF: Neutral detergent fibre
FPKM: Fragments per kilobase of exon per million fragments mapped
DEGs: Differentially expressed genes
FDR: False discovery rate
qRT-PCR: Quantitative reverse-transcription PCR
