# Supplementary figures and images for: Comparative transcriptomic analysis of seed coats with high and low lignin contents reveals lignin and flavonoid biosynthesis in Brassica napus
Source: BMC Plant Biol. 2021 May 29;21:246. doi: 10.1186/s12870-021-03030-5 (PMC8164251; doi:10.1186/s12870-021-03030-5)

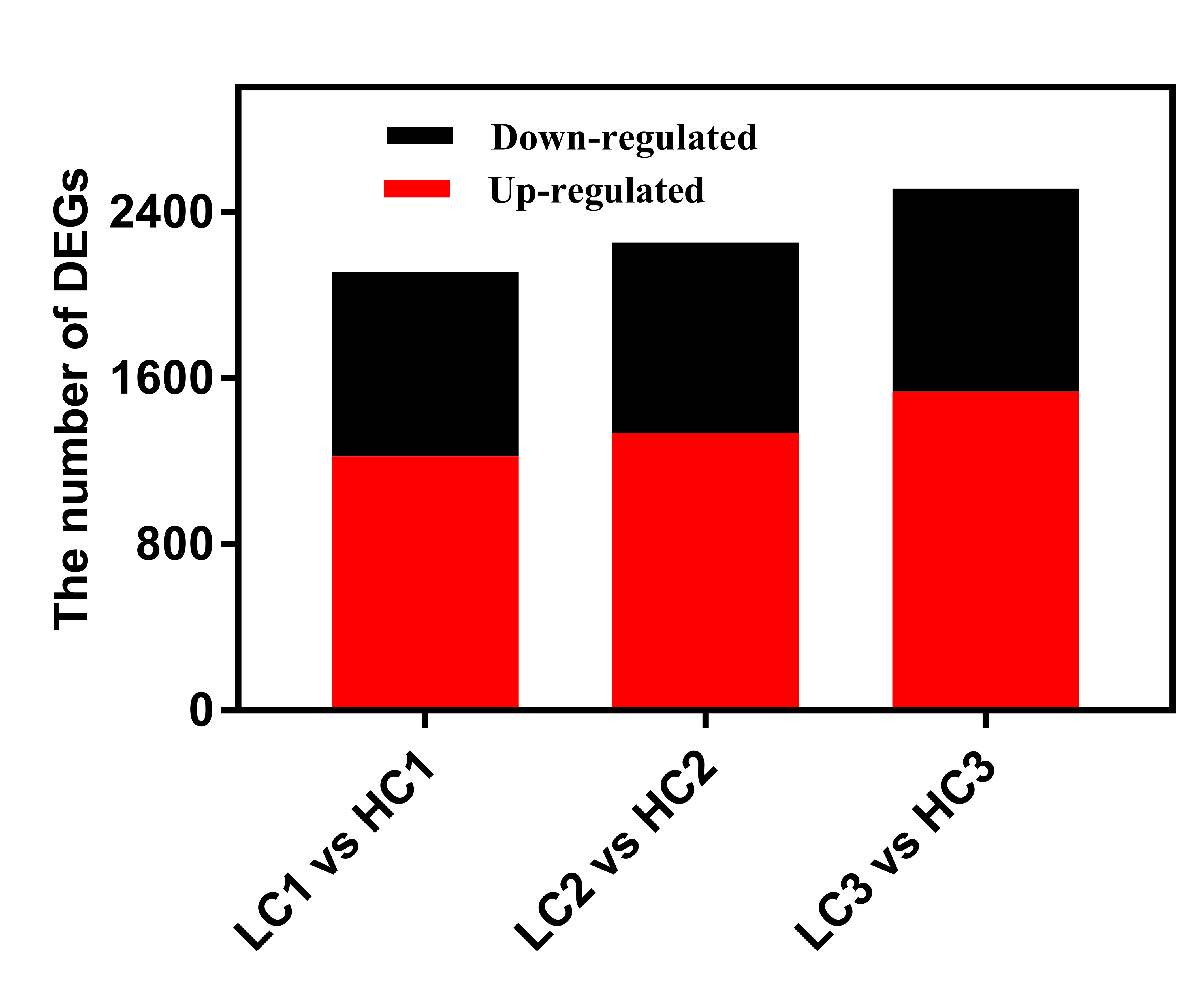

Supplement: Supplementary file 2 — Additional file 2: Fig. S1. Numbers of DEGs at three stages of seed coat development in H- and L-lignin lines. Red, upregulated; black, downregulated. [file 12870_2021_3030_MOESM2_ESM.tif]

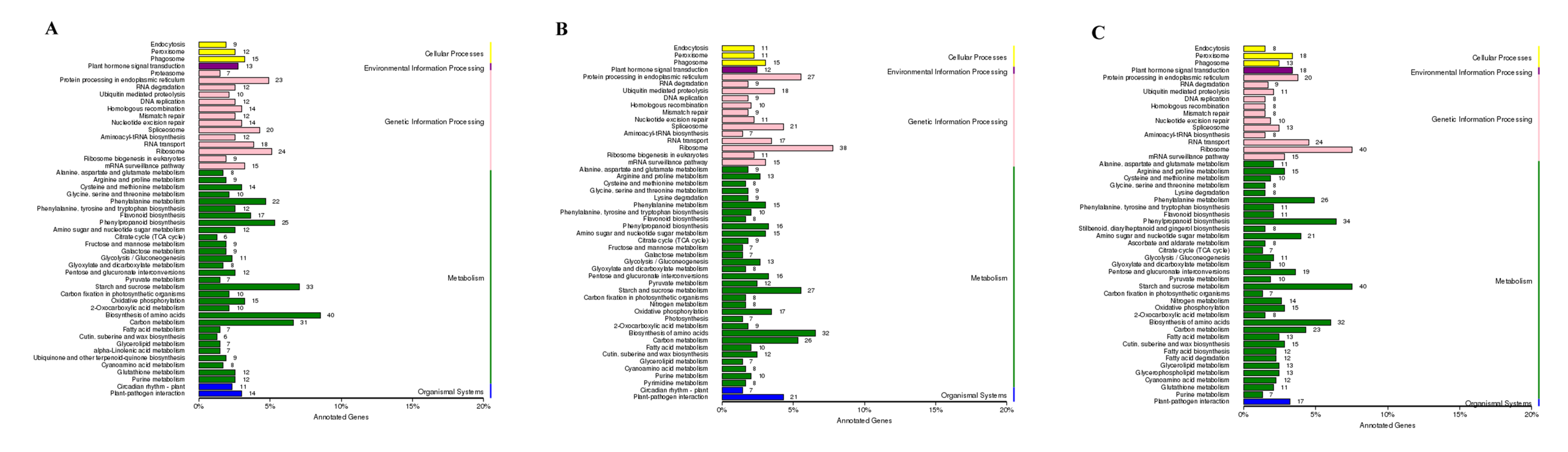

Supplement: Supplementary file 4 — Additional file 4: Fig. S3. KEGG analysis of DEG sets: (A) LC1 vs HC1, (B) LC2 vs HC2, and (C) LC3 vs HC3. [file 12870_2021_3030_MOESM4_ESM.tif]

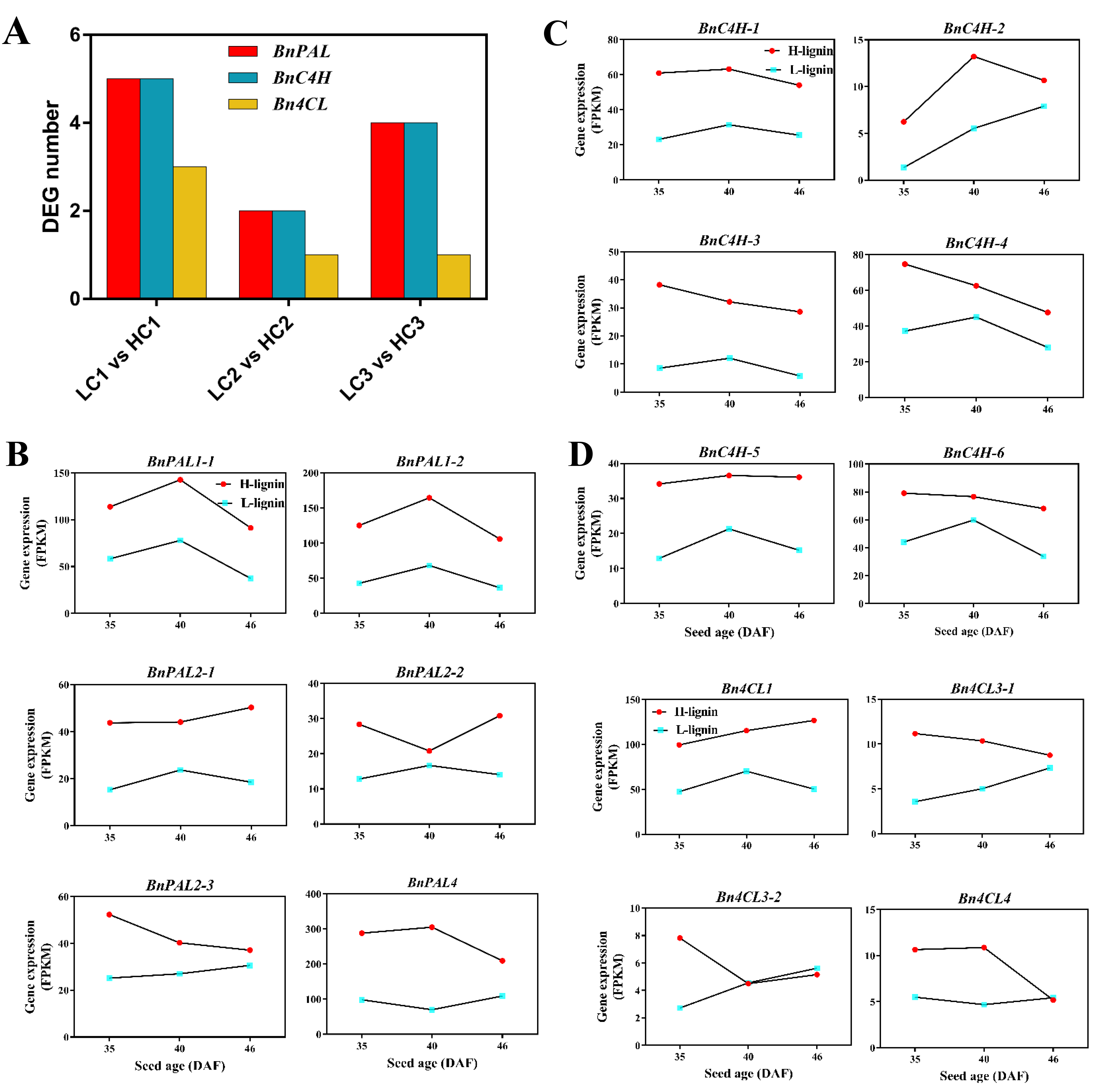

Supplement: Supplementary file 6 — Additional file 6: Fig. S5. Expression of DEGs encoding PAL, C4H, and 4CL in the phenylpropanoid pathway during three stages of seed coat development. (A) Number of DEGs in the BnPAL, BnC4H, and Bn4CL gene families. The expression (FPKM) of (B) BnPAL, (C) BnC4H and (D) Bn4CL gene families in three developmental stages of H- and L-lignin seed coats. [file 12870_2021_3030_MOESM6_ESM.tif]

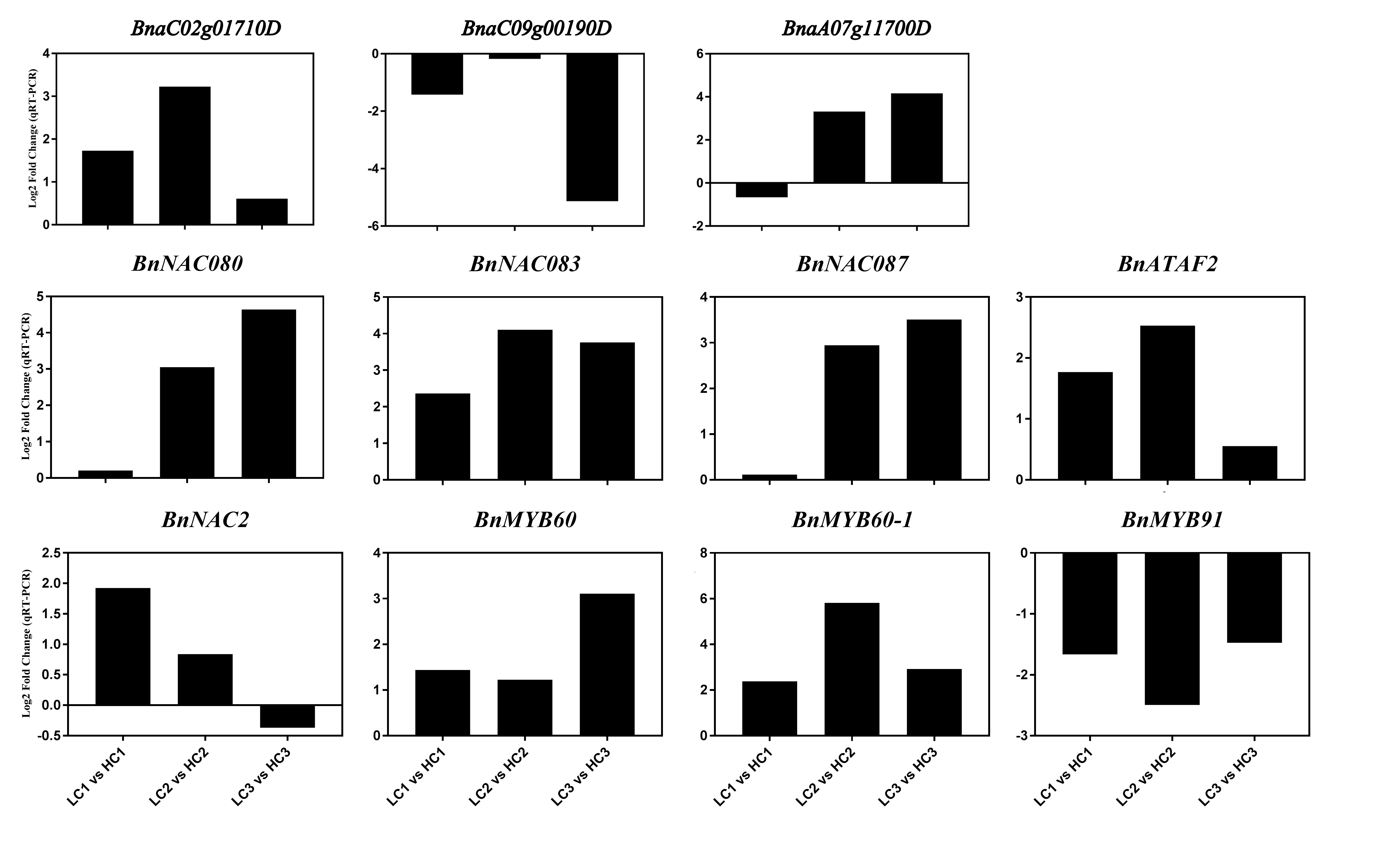

Supplement: Supplementary file 7 — Additional file 7: Fig. S6. Validation TFs and hormones related to lignin biosynthesis by qRT-PCR. [file 12870_2021_3030_MOESM7_ESM.tif]

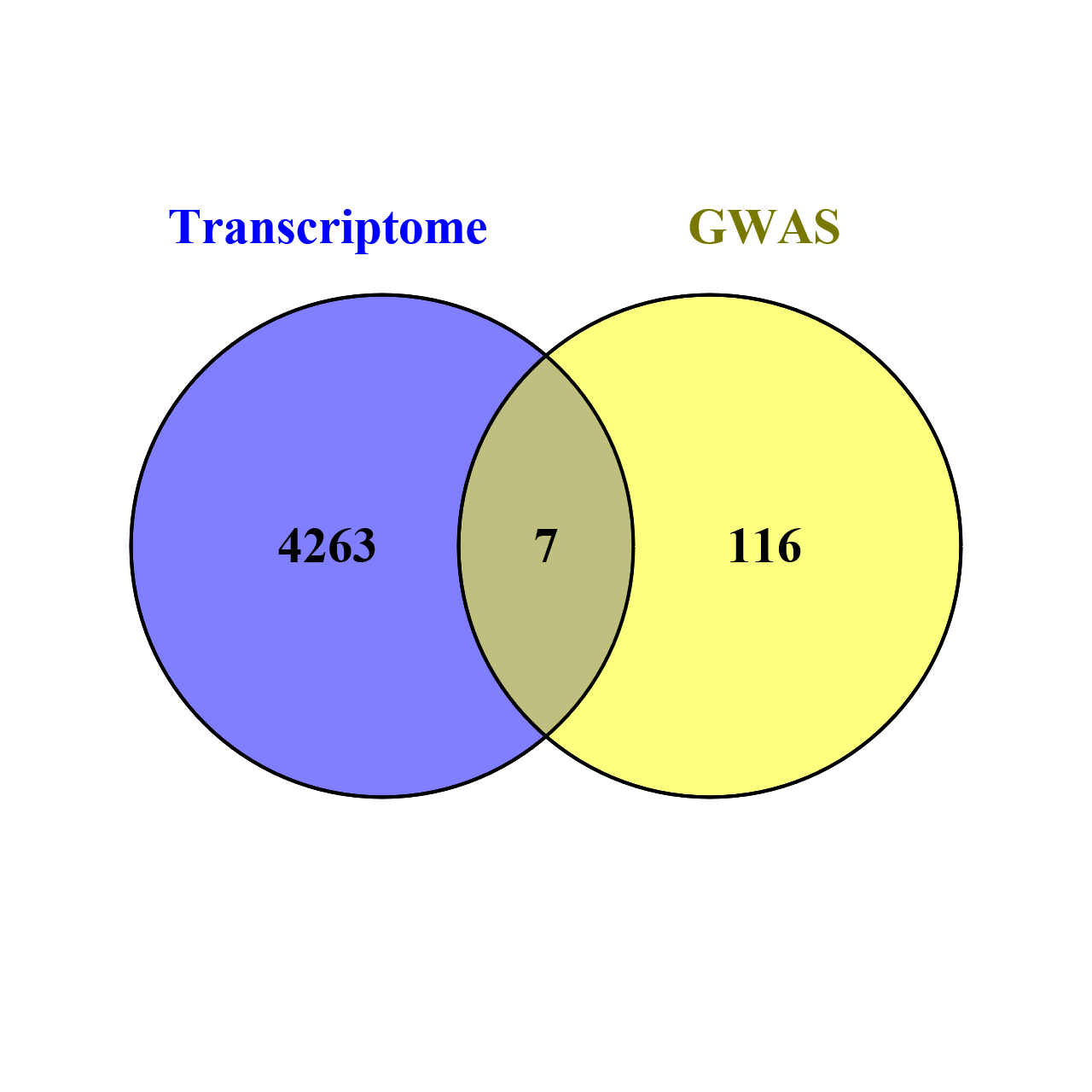

Supplement: Supplementary file 10 — Additional file 10: Fig. S9. Overlapping DEGs in the seed coat transcriptome and previous GWAS studies. [file 12870_2021_3030_MOESM10_ESM.tif]
